# Can computer-guided surgery help orthodontics in miniscrew insertion and corticotomies? A narrative review

**DOI:** 10.3389/froh.2023.1196813

**Published:** 2023-05-31

**Authors:** Rosanna Guarnieri, Camilla Grenga, Federica Altieri, Federica Rocchetti, Ersilia Barbato, Michele Cassetta

**Affiliations:** Department of Oral and Maxillofacial Sciences, School of Dentistry, “Sapienza” University of Rome, Rome, Italy

**Keywords:** CAD-CAM, digital orthodontics, surgical template, corticotomy, piezocision, miniscrew, TAD

## Abstract

Orthodontics has considerably increased the use of technology combined with surgery as a tool to improve dental movements in terms of predictability, acceleration of movement, and fewer side effects. To achieve these goals miniscrews and corticotomy were introduced. The digital workflow permits an increase in the accuracy of surgical and orthodontic setups. The tool that transfers the information is the CAD/CAM (*Computer-Aided Design/ Computer-Aided Manufacturing)* template. The aim of this review is to illustrate the use of computer-guided surgery in orthodontics regarding miniscrews and piezocision. The search strategy was a combination of Medical Subject Headings (Mesh) and free text words for PubMed. A total of 27 articles were included in this review: 16 concerned miniscrews and 11 concerned corticotomy. The current need for faster treatments, the improved systems of anchorage, and the evolution of imaging technologies require operators to be knowledgeable of the digital workflow. CAD/CAM templates allow greater precision and predictability of miniscrew insertion even if in the hands of less experienced clinicians and permit a better orientation and depth of the cortical incision. In conclusion, digital planning makes surgery faster and easier and allows for the identification and correction of any potential problem before the procedure.

## 1. Introduction

Throughout history orthodontics has faced several periods and, recently, has considerably increased the use of technology combined with surgery to support the improvement in dental movements in terms of predictability, acceleration of movement, and fewer side effects (1).

To achieve these goals, *TADs (Temporary Anchorage Devices)* were introduced to move the teeth in a more specific way, minimizing side effects, such as the loss of anchorage when not planned. In addition, *corticotomy interventions* began being used to speed up tooth displacement, thus allowing a much shorter treatment time (1, 2).

Moreover, radiographic techniques have evolved from conventional two-dimensional (2-D) radiographs to computerized tomography (CT) and cone-beam state of the computerized tomography (CBCT), allowing the skeletal and dental structures to be reconstructed in a detailed and three-dimensional way (3).

The successive advent of intraoral scanners introduced a three-dimensional (3-D) vision of the dental arches in STL format (an acronym for “*Standard Triangulation Language*”); these data in STL format can be matched or overlapped with the three-dimensional information of the CBCT using dedicated software (4, 5). The integration of all these digital information results in a digital workflow that permits more details and accuracy of surgical and orthodontic setups. Finally, the tool that allows clinicians to transfer the information of the digital workflow is the template designed and manufactured with the help of computer support (CAD/CAM, Computer Aided Design / Computer Aided Manufacturing) (6). The digital manufacturing technology related to 3-D printing allows customization of the surgical guide and concomitantly greater precision and predictability of the surgical procedure selected, as in the case of insertion of miniscrews or in corticotomy cuts (7-10).

### 1.1. Miniscrews

Orthodontic miniscrews provide skeletal anchorage, bypassing the patient's need for cooperation. For these reasons, their use has increased, as reported in several studies (11, 12). The boost in the use of miniscrews went hand in hand with the study of methods that would provide better stability during insertion, leading to greater success rates (3).

Surgical guides help clinicians to position dental implants with more accuracy and predictability. This also applies to mini-implants such as miniscrews or TADs in orthodontics (6).

### 1.2. Corticotomy

Corticotomy-accelerated orthodontics (CAO), based on the principle of regional acceleratory phenomenon (RAP), allows for a faster movement of the teeth. Dibart et al. introduced an innovative surgical technique to achieve rapid tooth movements while preserving periodontal support without extensive surgical trauma: the piezocision. Piezocision is a flapless procedure characterized by multiple small mucoperiosteal vertical incisions and ultrasonic corticotomies done with piezoelectric inserts (2).

The piezocision procedure, as well as the insertion of miniscrews, is greatly enhanced using a 3-D CAD/CAM surgical guide that minimizes the potential risks, such as incidental contact of tooth roots or any critical structure.

This is the first narrative review to illustrate the state-of-the-art use and the evolution in the use of computer-guided surgery in orthodontics relating to miniscrews and corticotomies. We aim to provide an overview and general indications of 3-D CAD/CAM technology both regarding the insertion of miniscrews and the surgical procedure for the acceleration of tooth movements. It is important to highlight how the 3-D CAD/CAM technological advance should not substitute but improve the diagnosis in orthodontics.

The need for this review is to illustrate the benefits of computer-guided surgery, a safe and affordable tool, in an era where digitization has become essential for everyday clinical practice.

## 2. Materials and methods

The literature search and study inclusion were carried out in duplicate by two review authors (R.G and C.G.). Any discrepancy was discussed with a third reviewer for consensus (M.C.).

### 2.1. Search strategy

The bibliographic research was carried out on Pubmed, MEDLINE (*via* Pubmed), EMBASE (*via* Ovid), Cochrane Reviews, and Cochrane Register of Controlled Trials (CENTRAL) from August 2022 until April 2023. The search strategy was a combination of Medical Subject Headings (Mesh) and free text words for PubMed and was optimized for each database. The details of the search strategy are summarized in Table 1. In addition, relevant journals and reference sections of retrieved studies were manually searched.

**Table 1:** Search strategies.

Search strategies	
1	CAD/CAM AND Orthodontics [Mesh]
2	Surgical template AND Orthodontics [Mesh]
3	Surgical guide AND Orthodontics [Mesh]
4	Piezosurgery [Mesh] AND Orthodontics [Mesh]
5	CAD/CAM AND corticotomy
6	Surgical guide AND corticotomy
7	Surgical template AND corticotomy
8	Computer Aided Design [Mesh] AND piezocision
9	Surgical guide AND piezocision
10	Surgical template AND piezocision
11	Surgical guide AND miniscrew
12	Surgical guide AND TADs
13	Surgical template AND miniscrew
14	Surgical template AND TADs

### 2.2. Included study types

Randomized controlled trials (RCTs), quasi-RCT (Q-RCTs), controlled clinical trials (CCTs), unclear non-randomized studies (uNRS), prospective and retrospective studies, systematic reviews with and without meta-analysis, reviews, and case reports were included in this study. We decided to include case reports considering the small number of other kinds of studies relevant to the research topic. Eligibility criteria comprised articles published in English after 1990, and those that included a description of computer-guided surgery in orthodontics and a description of the digital workflow of computer-guided surgery in orthodontics. Exclusion criteria were: non-English language studies and FEM/laboratory/animal studies. Thus, studies dealing with the surgical template in the context of mini-screws and piezocision were analyzed.

### 2.3. Study selection

A total of 1,795 studies were identified through the database search. Out of these, 863 were excluded as duplicates. Abstracts were read and 859 articles were excluded because they were not related (irrelevant) to the research that was being carried out.

**Figure 1:**
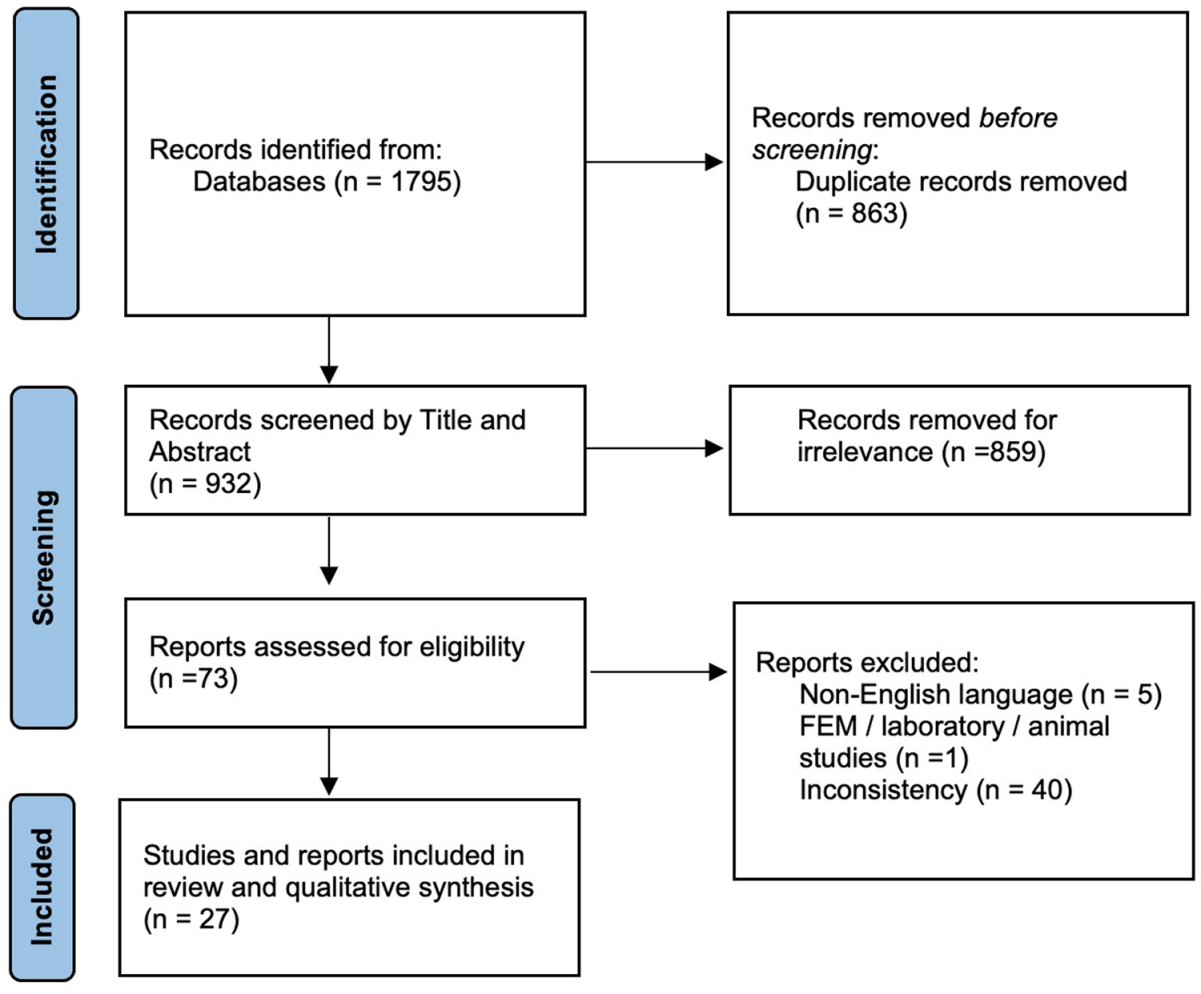
Flowchart of the study selection.

The full texts of the remaining 73 articles were examined and 45 were excluded because they did not match the eligibility criteria: 5 of them were excluded because the full text was not in English, 40 studies were inconsistent with respect to the purpose of the study and 1 was based on animal tests. Finally, 27 articles were included in this review and qualitative synthesis. The selection procedure is represented in Figure 1.

### 2.4. Relevant sections

Of the 27 studies analyzed on computer-guided surgery, 16 concerned miniscrews and 11 concerned piezocision. Of these 27 studies, one was an RCT, four were CCTs, five were prospective studies, two were retrospective studies, one was a review, three were systematic reviews, and one was a systematic review with meta-analysis. Finally, there were 10 case reports. The data are summarized in Table 2. The qualitative synthesis then envisaged the structuring of two major paragraphs or relevant sections.

**Table 2:** Study types.

Authors	Section	Type of study	Content of the study
Bae M et al. (3)	Miniscrew	CCT	To evaluate the accuracy of miniscrew placement by using surgical guides developed with computer-aided design and manufacturing techniques. Miniscrews were placed more accurately when using surgical guides than when using a direct method.
Liu H et al. (4)	Miniscrew	CCT	To enable accurate miniscrew placement after preoperative simulation with computed tomography, developing a new template for miniscrew placement and evaluating its accuracy. The proposed template has high accuracy and will be especially useful for patients who require precise miniscrew placement.
Suzuki E et al. (6)	Miniscrew	Retrospective Study	To assess the accuracy of miniscrew placement into the dentoalveolar bone, aided by a 3-dimensional (3D) surgical guide, and to compare the results with those from conventional procedures. The 3D surgical guide provides a precise method for miniscrew placement into the dentoalveolar bone. The accurate insertion of miniscrews using the 3D surgical guide allows orthodontists to precisely transfer the radiographic information from preoperative planning to the surgical site, thus minimizing the risks of root injury.
Miyazawa K et al. (8)	Miniscrew	CCT	To avoid possible root damage during miniscrew insertion, a surgical guide was fabricated. CBCT images of post-surgical self-drilling miniscrew implant placement showed no root contact. The use of surgical guides, fabricated using CBCT images, appears to be a promising technique for the placement of orthodontic self-drilling miniscrew implants adjacent to the dental roots and maxillary sinuses.
Qiu L et al. (13)	Miniscrew	CCT	To develop surgical stents for CBCT 3D image-based stent-guided orthodontic miniscrew implantation and to evaluate its accuracy. 3D CBCT image-based SLA-fabricated surgical stents can provide a safe and accurate method for miniscrew implantation. 3D CBCT image-based SLA-fabricated surgical stents can provide a safe and accurate method for miniscrew implantation.
Cassetta M et al. (14)	Miniscrew	Case Report	To describe a new computer-guided technique for controlled site preparation and palatal orthodontic miniscrew insertion using dedicated software. The present surgical guide allows a controlled and accurate palatal miniscrew placement in three dimensions.
Altieri F et al. (15)	Miniscrew	Case Report	To present a minimally invasive approach to solving the impaction of palatal canines using computer-guided orthodontic miniscrews. The use of computer-guided skeletal anchorage allows for both the reduction of the biomechanical side effects typical of conventional treatment and the risk of damaging adjacent anatomical structures, increasing the effectiveness of treatment.
Lo Giudice A et al. (16)	Miniscrew	Systematic Review	To evaluate the complications and side effects associated with the clinical use of orthodontic miniscrews by systematically reviewing the best available evidence. Findings highlight the need for clinicians to preliminarily assess generic and specific insertion site complications and side effects.
Park HS et al. (17)	Miniscrew	Retrospective Study	To provide information about placement sites and angulations of microimplants between tooth roots, analyzing 3-dimensional computed tomography images from 25 patients. To minimize root contacts, microimplants need to be inclined distally approximately 10 degrees to 20 degrees and placed 0.5 to 2.7 mm distally to the contact point to minimize root contact according to sites and levels, except into palatal interradicular bone between the maxillary first and second molars.
Lo Giudice A et al. (18)	Miniscrew	Case Report	To evaluate bone characteristics, choosing the most suitable location for miniscrew insertion and the design of a fixed positional template in the 3D printed maxillary model. The workflow is illustrated along with a documented case report.
D’Haese J et al. (19)	Miniscrew	Review	To summarize the evolution and ongoing trends in digital and virtual planning and in implant surgery. To clarify the different concepts in guided surgery and their respective advantages, disadvantages, and limitations. The outcome of guided surgery is assessed in terms of implant survival, precision, and complications. Clinical cases are given to demonstrate briefly the workflow and clinical guidelines for the safe use of these approaches.
Cassetta M et al. (20)	Miniscrew	Prospective Study	To assess whether there is a learning curve in static computer-assisted implant surgery (s-CAS). A typical “learning curve” effect was not identified for s-CAS.
Sánchez-Riofrío D et al. (21)	Miniscrew	Case Report	To describe a titanium grade V computer-aided design/computer-aided manufacturing (CAD/CAM) maxillary expander supported by two miniscrews, along with a 3D printed surgical guide. Digital models, CBCT, and CAD/CAM technology are essential to accomplish the goals proposed in this article. Further studies are necessary to establish safer miniscrew placement sites and insertion angles to achieve greater in-treatment stability. Both the clinician and the patient can benefit from the use of current technology, creating new devices and updating traditional orthodontic procedures.
Su L et al. (22)	Miniscrew	Prospective Study	To analyze the structure of the infrazygomatic crest and 3D printing technology, develop two kinds of templates, and evaluate their clinical effects. In the vertical direction, the accuracy of implantation with the template is higher than that of the traditional method without the template to avoid piercing the maxillary sinus mucosa in the infrazygomatic crest zone.
Kniha K et al. (23)	Miniscrew	Prospective Study	To evaluate the accuracy of fully guided orthodontic mini-implant (OMI) placements supported by tooth- (TBGs) or gingiva-borne silicone guides (GBGs) based on virtually superimposed lateral cephalograms on virtual plaster models. The accuracy of an OMI position can be significantly increased by using a guide extension over the teeth.
Kernen F et al. (24)	Miniscrew	Prospective Study	To test whether digitally designed three-dimensional printed templates (D-temp) fabricated by matching surface scans and cone beam computed tomography (CBCT) images differ from the templates fabricated in-lab (L-temp) by using a physical transfer device for the positioning of the guiding sleeves. Higher accuracy of implant placement can be achieved by using three-dimensional printed templates produced by matching a surface scan and CBCT as compared with templates that use physical elements to transfer the virtual planning into reality.
Long H et al. (25)	Piezocision	Systematic Review	To evaluate the effectiveness of interventions on accelerating orthodontic tooth movement. Corticotomy is effective and safe to accelerate orthodontic tooth movement, low-level laser therapy was unable to accelerate orthodontic tooth movement, current evidence does not reveal whether electrical current and pulsed electromagnetic fields are effective in accelerating orthodontic tooth movement, and dentoalveolar or periodontal distraction is promising in accelerating orthodontic tooth movement but lacks convincing evidence.
Dibart S et al. (26)	Piezocision	Case Report	To introduce a new, minimally invasive procedure, combining microincisions with selective tunneling that allows for hard- or soft-tissue grafting and piezoelectric incisions. This novel approach is leading to short orthodontic treatment time, minimal discomfort, and great patient acceptance, as well as enhanced, or stronger, periodontium.
Yi J et al. (27)	Piezocision	Systematic Review	To evaluate the effect of piezocision as an adjunctive procedure to accelerate orthodontic tooth movement. Based on currently available information, weak evidence supports that piezocision is a safe adjunct to accelerate orthodontic tooth movement, at least in the short term. More high-quality clinical trials to determine the long-term effects and optimal protocol for piezocision are needed to draw more reliable conclusions.
Mheissen S et al. (28)	Piezocision	Systematic Review with metanalysis	To critically assess the overall effectiveness of Piezocision in accelerating orthodontic tooth movement, as well as the adverse effects of this intervention in orthodontic patients. Low-quality evidence suggests that piezocision is an effective surgical procedure in accelerating the rate of canine retraction in the first two months and reducing the treatment duration. However, this effect appears to be clinically insignificant.
Milano F et al. (29)	Piezocision	Case Report	To present a method for combining piezocision with the use of computed tomography. By creating a three-dimensional model of the arch, the depth, and location of the corticotomies can be precisely planned, and a surgical guide can be fabricated and used to prevent any damage to the dental roots.
Cassetta M et al. (30)	Piezocision	Case Report	To overcome the disadvantages of the corticotomy, this technical note describes an innovative, minimally invasive, flapless procedure combining piezoelectric surgical cortical micro-incisions with the use of a 3D Printed CAD/CAM surgical guide.
Cassetta M et al. (31)	Piezocision	Prospective Study	To evaluate the effectiveness of an innovative, minimally invasive, flapless corticotomy procedure in orthodontics. This new procedure is safe and accelerates tooth movement without periodontal complications or discomfort.
Cassetta M et al. (32)	Piezocision	Case Report	To describe an innovative orthodontic treatment method that combined surgical and orthodontic techniques. The reduction in surgical time and patient discomfort, increased periodontal safety and patient acceptability, and accurate control of orthodontic movement without the risk of losing anchorage may encourage the use of this combined technique in appropriate cases.
Hou HY et al. (10)	Piezocision	Case Report	To present a novel 3-dimensional (3D) computer-assisted piezocision guide (CAPG) designed to be translucent for increased visibility, rigid for enhanced support during guidance, and porous for profuse irrigation during the procedure. This minimally invasive procedure was uneventful, and no devitalized tooth or alveolar bone was found.
Gibreal O et al. (33)	Piezocision	RCT	The aim of this trial was to assess computer-guided piezocision-based orthodontics. The values of the surgical guide deviation were nearly null, which confirms that this innovative technique is clinically applicable. Furthermore, this technique was impressively effective in accelerating orthodontic tooth movement.
Cassetta M et al. (34)	Piezocision	Case Report	To provide detailed information on the design and manufacture of a 3D-printed CAD-CAM (computer-aided design and computer-aided manufacturing) surgical guide that can aid the clinician in achieving a minimally-invasive, flapless corticotomy. The effectiveness of this minimally-invasive surgical technique can offer the clinician a valid alternative to other methods currently in use.

## 3. Miniscrews for orthodontic anchorage

Anchorage is one of the essential elements for the success of orthodontic treatment. Traditionally, the teeth themselves or appliances have been used as intra and extraoral anchors, often relying on the patient's compliance for the success of the treatment (35). TADs are compliance-free and provide absolute anchorage during orthodontic treatment both directly and indirectly (13).

 Currently, among the TADs, the most used anchoring devices are the miniscrews, which are positioned in the midsagittal area of the palate, in the retromolar area of the mandible, and also in the dentoalveolar area thanks to the reduced size of the diameter that allows the insertion between the roots of adjacent teeth (4). Miniscrews are relatively inexpensive, easy to insert and remove, versatile (they can be positioned in different areas of the maxilla and mandible), and, above all, they are predictable enough to be used routinely in clinical practice (14). The insertion areas can be interradicular and extra-radicular. Among the extra-radicular sites, the tuberosity of the maxilla, the zygomatic process, the chin region, the buccal shelf, and more frequently the anterior palate can be identified as areas of interest. The third palatine ruga is a reliable clinical reference point for assessing bone availability and thickness for the placement of miniscrews in the anterior palate (36-38).

Nowadays, miniscrews are widely used to improve the efficiency of dental movements (mesialization-distalization, intrusion-extrusion, retraction, and uprighting) decreasing the possible side effects of conventional treatments (15). Nevertheless, the clinical use of miniscrews is not free from potential unwanted effects and complications that may occur during insertion, use, and removal.

A complication that can occur during the insertion and removal of miniscrews, especially in the posterior region and in areas of very dense bone, is fracture or breakage of the miniscrew. The tendency to change the insertion angle by moving the handpiece or increasing torsional stress can lead to a fracture of the neck of the miniscrew (16). However, the most frequent complication reported in the literature is root damage during the interradicular insertion of the miniscrew. This complication can also occur during the insertion of the miniscrews in the anterior area of the palate. The most frequent side effects are pain, inflammation of soft and hard tissues, and hypertrophy of the peri-implant gingival tissue (3, 16). The miniscrew-tooth contact, and the lesion of the root, which can be of varying degrees of severity up to the loss of vitality of the dental element, can contribute to the early loss of the miniscrew. For these reasons, the insertion of miniscrews requires appropriate anatomical knowledge and radiographic evaluation to guide the clinician in determining the safest area for insertion. Regarding interradicular miniscrews, some authors recommend a positioning from 4 to 6 mm under the alveolar ridge with an angle from 30° to 45 ° with miniscrews that have a diameter between 1.2 and 1.6 and a length of 6-8 mm (3, 17). A good compromise in the use of interradicular miniscrews may be to consider an average miniscrew diameter of 1.5 mm and a minimum interradicular distance of 3 mm (39). Different methods have been proposed in order to achieve a precise and safe positioning for interradicular miniscrews. For example, the use of a radiopaque reference in an endoral 2D x-ray has been proposed in order to assess the mesiodistal distance of the roots (40). These methods based on two-dimensional image evaluation, however, cannot guarantee predictable and precise positioning due to the absence of three-dimensional programming and control in the miniscrew positioning (3).

A controllable, reliable, and reproducible method for miniscrew positioning is important for orthodontists and, nowadays, a CAD/CAM surgical guide can be a viable solution (2). CAD/CAM technology has been used in implantology to determine accurate positioning and to manufacture surgical guides for dental implant insertion. Computed tomography or Cone Beam Computer Tomography (CT or CBCT) is performed to assess bone quality and quantity, and implant position is determined based on 3D digital images that are reconstructed from CT or CBCT data (18).

It should be noted that the reconstruction of digital 3D images from computer tomography images alone is associated with image distortion of computed tomography or artifacts caused by metal in the mouth. To overcome these problems, it has been proposed and suggested to combine images of the dental arch, including both scanned images of plaster models and intraoral scanning data, with images of computed tomography (19).

**Figure 2:**
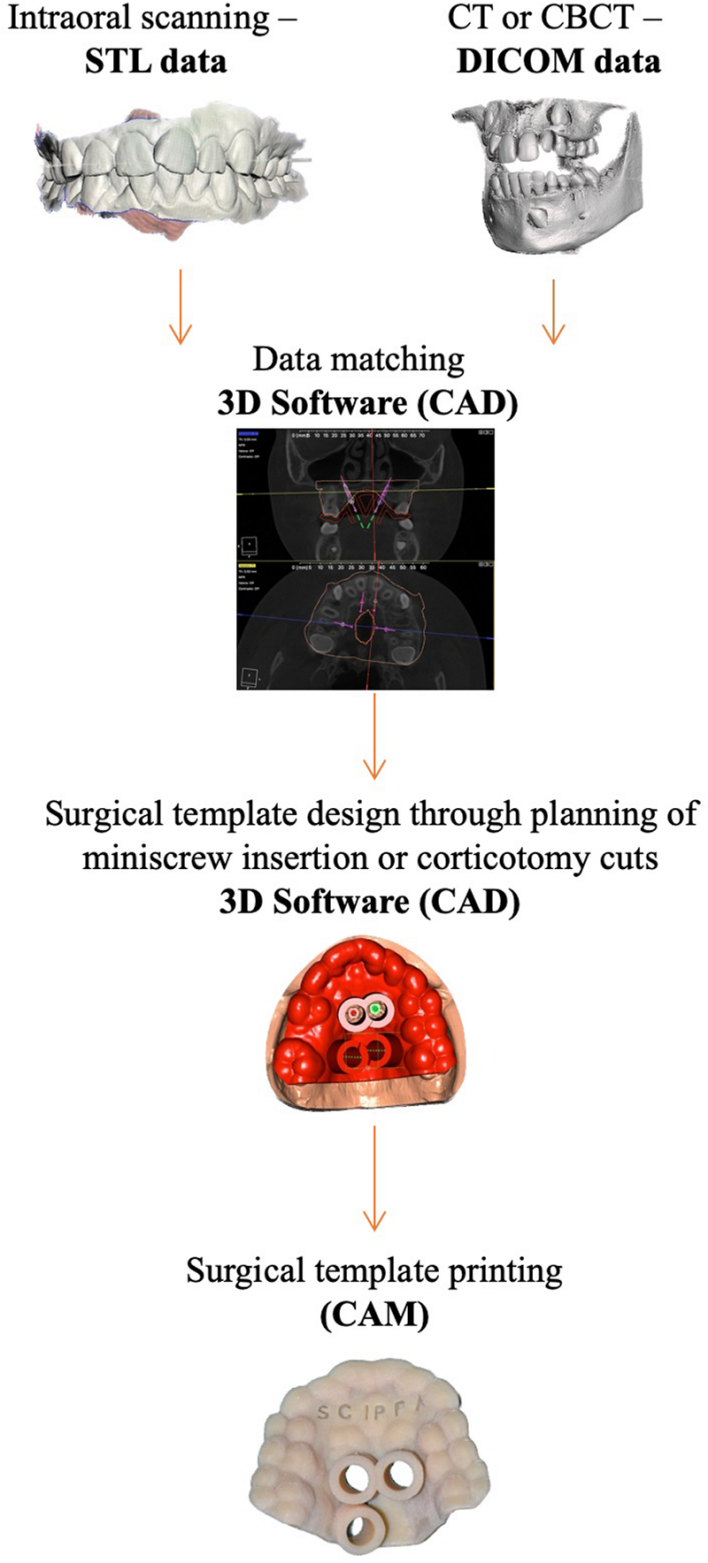
The workflow of computer-guided surgery in miniscrews insertion and corticotomies.

The surgical guide is built starting from the STL files of the arches and the digital model images are fused with CT or CBCT images of the jaws using 3D software. On the STL 3D model file the ideal points for miniscrew insertion are identified and a software application is used for the design of the surgical guide. Then the 3D STL model of the surgical guide is printed using a 3D printer through stereolithography apparatus (SLA). SLA is the process of using laser-cured photosensitive resins layer by layer (2). A diagram of the digital workflow is summarized in Figure 2.

The CAD/CAM technology allows the clinician to design and plan everything in a single visit, while the 3D printed surgical guide allows the positioning of the miniscrews or the miniscrews together with the device on the same day, confirming their precise position according to the digital planning. The accuracy of this technique is discussed by Cassetta et al. and Sánchez-Riofrío et al. who highlighted that it lacks a “learning curve” effect, thus making it a simple procedure with a very low risk of failure (20, 21). CAD/CAM technology can therefore help less experienced clinically-surgical operators in safely positioning the miniscrews. Several studies show how the use of surgical templates allows bicortical anchorage during TADs insertion, improves primary stability, and reduces the risk of injury to contiguous anatomical structures (16, 18).

Qiu et al. compared the precision of insertion of miniscrews between a study group where insertion was computer guided and a control group where insertion was freehand; the results showed that no root had been touched with computer-guided insertion while in the freehand group, some miniscrews had come into contact with the root. The same study also demonstrated how the programmed insertion accuracy was highly more reliable in the study group than in the control group (13). Another study by Su et al. confirmed that the use of CAD/CAM templates can increase the accuracy of inserting miniscrews, thus reducing the risk of damaging adjacent structures (22). Similar results were reported by Suzuki et al. (4). In addition, Bae et al. observed that in the study group with guide placement of miniscrews, no miniscrews came into contact with the roots of the adjacent teeth (3); Miyazawa et al. found that the success rate of miniscrew placement was 90.9% when surgical guides were used (6).

The interradicular area of the maxilla is often too small for the placement of miniscrews, leading to the infrazygomatic crest being proposed as an alternative. The infrazygomatic crest has a double cortical layer and is close to the center of resistance of the maxilla, providing greater anchorage. However, it is adjacent to the maxillary sinus and the apical portion of the roots and the risk of injury is present. Therefore, the template is also very useful in cases of insertion into the infrazygomatic crest (22).

Surgical guides for TADs insertion can be manufactured in acrylic resin or in PMMA (Polymethyl methacrylate) and are anchored to the teeth mechanically or chemically. Depending on the characteristics (stiffness) of the template material, the retention tooth cover can be complete or partial with the addition of any retention hooks. The guide must be firmly anchored in order to avoid unwanted movement during the insertion of the miniscrews. In fact, many parameters potentially contribute to the accuracy of the insertion of the computer-guided miniscrews: radiographic inaccuracies, intraoral scanning, the production of the plasters, and surgical guide 3D printing (23, 24).

The insertion accuracy also depends on the transfer accuracy: generally, surgical guides can be supported by teeth, mucosa, bone, and implants, but for TADs insertion, dental support is preferable (16). Movements of the surgical guide might cause differences in the deviation during implant site preparation, for this reason, it is preferable to use sleeves (metal or polymeric material) inserted in the surgical template in order to direct the drill more precisely and without the risk of potential deviations during perforation (23).

In the placement of palatal miniscrews in deep and narrow palatine vaults, one difficulty that must be overcome is the convergence of the insertion axes of the miniscrews. In these cases, in order to achieve good insertion accuracy, two surgical templates are required to prevent the insertion handpiece from coming into contact with the template itself.

The use of surgical guides built with CAD/CAM technology is an important tool in the hands of a less experienced clinician and serves as an encouragement for the initial use of the miniscrews. In the hands of more experienced clinicians, surgical guides built with CAD/CAM technology increase the predictability of insertion (18).

## 4. Corticotomy and piezocision

The growing demand from adult patients for orthodontic treatment determined the development and the consequent evolution of methods and alternatives able to shorten the duration of the treatment. The absence of growth in adult patients also involves different therapeutic approaches: the phenomena of hyalinization and reduced cell mobilization during treatment are more frequent in subjects at the end of growth, and a lower conversion rate of the collagen fibers and an increased probability to develop periodontal complications are usual (41).

In a systematic review by Long et al. several novel modalities were reported to accelerate orthodontic tooth movement, including low-level laser therapy, pulsed electromagnetic fields, electrical currents, mechanical vibration, distraction osteogenesis, and corticotomy (42). The review showed that corticotomy is only effective and safe in accelerating orthodontic tooth movement (25). Other more recent reviews confirmed the effectiveness of corticotomy in accelerating orthodontic movement (43–45).

In the first decade of this century, Wilcko et al. proposed the corticotomy approach to accelerate orthodontic tooth movement. They called this method Accelerated Osteogenic Orthodontics (AOO) technique or, more precisely, Periodontally Accelerated Osteogenic Orthodontics (PAOO) technique (46). The technique consists of full-thickness flaps labially and lingually inserted in the zone in which the teeth will be moved. Then circumferential corticotomy cuts and intramarrow penetrations are made with rotating burs. Finally, the “activated” bone is covered with a particulate bone grafting mixture and the flaps are sutured to completely cover the surgical area. This procedure is based on the concept of regional acceleratory phenomenon (RAP) defined by Frost: immediately after corticotomy, during the healing process, there is transitory osteopenia responsible for the rapid tooth movement (47).

Many studies, also on experimental animals, have successively illustrated the effectiveness of the corticotomy technique in terms of shortening treatment time (48–51). The acceleration of the orthodontic tooth movements immediately after corticotomy seems to be attributed to increased bone turnover due to a greater number of osteoclasts in the stimulated area. In 2019, a review of the literature stated that alveolar corticotomies as an adjunct to orthodontic treatment can decrease total treatment time for the orthodontic patient (52).

In 2007, Vercellotti et al. and, in 2011, Bertossi et al. proposed a new surgical approach to the corticotomy: the use of a piezosurgical device to minimize the side effects of the traditional burs, so reducing the traumatism at the bone and ensuring better cutting precision, more predictable bone regeneration, and faster healing with minimal morbidity (53, 54). Dibart further perfected this technique, making it easier and less traumatic by removing the elevation flap. The technique was called Piezocision, which means a corticotomy performed with a piezosurgical device with or without bone graft and without flap elevation. Sutures are not required if a bone graft is not performed (26).

In a study conducted on rats in 2016, Han and He stated that the use of piezosurgery for the corticotomy used in accelerating the movement of the teeth during orthodontic treatment is associated with an increase of the bone morphogenetic protein-2 (BMP-2) (55). BMP-2 is a multifunctional growth factor that promotes the proliferation, differentiation, and apoptosis of many cells and is involved in the regeneration and repair of tissues, particularly bone tissues.

A systematic review, conducted in 2017, concerning the efficacy of piezocision on accelerating orthodontic tooth movement, stated that weak evidence supports that piezocision is a safe adjunct to accelerate orthodontic tooth movement and that more high-quality clinical trials are needed to draw more reliable conclusions (27). Furthermore, a systematic review with meta-analysis published in 2020 suggested that piezocision is an effective surgical procedure in accelerating tooth movement and in reducing the treatment duration even if further studies are needed (28).

The surgical cut of the bone is performed in an area of close root proximity and this is a potential risk for root damage. Moreover, corticotomy during piezocision is carried out flapless making it even more difficult to correctly identify the dental roots and their root tips.

During these last years, the introduction of more refined diagnostic techniques, such as the CBCT, has allowed a 3D reconstruction of the maxilla and the mandible, permitting very precise planning of the depth and location of the decortications. It is possible to transfer the CBCT data to a 3D printer in order to create a surgical guide that assures a more predictable flapless cut without potential risks to the dental root, which also reduces surgical treatment time. In 2014, Milano et al. published a case report illustrating a method for combining piezocision and CBCT to construct a surgical guide to make high-precision bone cuts. By creating a three-dimensional (3D) model of the arch, the depth and location of the corticotomies were planned and transferred to a resin surgical guide using a numerically controlled milling machine (29). Cassetta et al. successively illustrated in 2015, 2016, and 2017, as well as Hou et al. in 2019, the procedures to perform a 3D computer-assisted surgical guide (30–33). Finally, in a randomized controlled clinical trial, Gibreal et al. confirmed that computer-guided surgical templates are effective and clinically applicable tools (33).

First of all, the impressions must be taken with extreme precision trying to go as far as the reading of the fornix in order to then obtain adequate templates for the length of the corticotomy lines. The presence of orthodontic brackets during the impression could create inaccuracies for the subsequent construction of the template, but today this problem is solved by the intraoral scanner and the development of a digital model (5). During the impression phase, attention must be paid to the relief of the frenulum which could then complicate the correct insertion of the surgical guide (7). Furthermore, the use of transparent material to construct the surgical guide can facilitate a direct view of the operating field (8). To avoid damage to the mucosal tissue and alveolar bone, due to overheating during the computer-guided procedure, multiple pores and drain tubes can be performed on the surgical guide to allow copious saline irrigation to have greater access to the surgical guide (31, 34).

In addition, it is important to highlight how the use of CBCT in the planning phase of the intervention is an important tool in the identification of vascular-nervous structures, thus decreasing the risk of damage to these structures through the CAD/Cam template (56).

A limitation of corticotomy and piezocision is the additional surgery the orthodontic patient has to undergo; furthermore, this technique cannot predictably move ankylosed teeth or be performed in devitalized bone, a frequent situation in patients being treated with bisphosphonates or corticosteroids (57).

In conclusion, the use of a surgical guide during corticotomy and the piezocision technique can permit a better and more precise orientation and depth of the cortical incision. With the aid of a CBCT, it is also possible to perform cuts in areas where the cortical bone is thick, avoiding damage to the root of the teeth and limiting postoperative discomfort and complications.

## 5. Limitations, complications, and contraindications of computer-guided surgery

Computer-guided surgery is not without its criticalities. It is always necessary to consider both the additional biological cost in the prescription of a second-level investigation such as CBCT and the possibility of complications that would not otherwise occur, such as the breakage of the surgical guide itself during its use (16). Considering the use of miniscrews in pediatric patients we refer to the DIMITRA project for indication-oriented and patient-specific recommendations regarding the main cone-beam CT applications in the pediatric field (58). Last but not least, a careful examination of the patient's condition is always necessary: a reduced mouth opening is a contraindication to the use of surgical templates due to the increased size of the mounting device with regard to the height of the surgical guide tube.

## 6. Limitations, perspectives, and strengths of the study

This is the first narrative review that offers an overview of computer-guided surgery for miniscrews and corticotomy in orthodontics using a standardized methodology and reporting on its practical applications and advantages. However, this review does not allow for analytical data. To date, there are few RCT, CCT, and very heterogeneous studies, which do not allow for a systematic review. It would be interesting to conduct studies with a standardized methodology and then conduct meta-analyses on the collected data and offer the reader an objective report of the results, for example with regard to the operator's comfort or not in using the computer-guided surgical template in orthodontics.

In addition, not having included studies in another language may have marred the quality of the work.

## 7. Conclusions

The current need for faster treatments, the improved systems of anchorage, and the evolution of imaging technologies for accurate diagnosis and treatment simulation require orthodontists to be knowledgeable of the digital workflow. The use of CAD/CAM templates allows greater precision and predictability of the surgical technique selected for the purpose of personalized orthodontic treatment. In this way it is possible to reduce the risk of injury to contiguous anatomical structures and to reduce surgical treatment time, allowing better comfort to the patient. This planning makes surgery faster and easier and allows any potential problems to be identified and corrected before the procedure.
